# The Development of a Hoof Conformation Assessment for Use in Dairy Goats

**DOI:** 10.3390/ani9110973

**Published:** 2019-11-14

**Authors:** Laura E. Deeming, Ngaio J. Beausoleil, Kevin J. Stafford, James R. Webster, Maryann Staincliffe, Gosia Zobel

**Affiliations:** 1 AgResearch Ltd., Ruakura Research Centre, 10 Bisley Road, Hamilton 3214, New Zealand; laura.deeming@agresearch.co.nz (L.E.D.); jim.webster@agresearch.co.nz (J.R.W.); maryann.staincliffe@agresearch.co.nz (M.S.); 2 School of Veterinary Science, Massey University, Palmerston North 4442, New Zealand; N.J.Beausoleil@massey.ac.nz (N.J.B.); K.J.Stafford@massey.ac.nz (K.J.S.)

**Keywords:** toe length, heel shape, claw splay, claw shape, subjective scores, objective measures, lameness, welfare

## Abstract

**Simple Summary:**

In comparison to other species, there are little data evaluating hoof conformation in dairy goats. As poor conformation is associated with an increased risk of hoof lesions and lameness, it is important to be able to accurately and reliably assess hoof conformation. This study developed a reliable hoof conformation assessment for use in dairy goats using hoof photographs. The assessment included both subjective scores and objective measures. High levels of accuracy were achieved when comparing two aspects of the subjective scores against two objective measures. This suggests the subjective scores may be a suitable alternative to the more time-consuming objective measures.

**Abstract:**

The assessment of hoof conformation is important due to its recognized relationship with the biomechanical functionality of the hoof. Hoof conformation can be assessed using objective measures or subjective scores. However, to date, there are limited data using either method in dairy goats. Therefore, the aims were to (1) develop a reliable method of assessing hoof conformation in dairy goats, and (2) compare two aspects of a subjective assessment against corresponding objective measures as a means of validation. A total of 1035 goats contributed photographs across 16 commercial dairy goat farms. Photographs were taken of the left front and left hind hoof in the lateral and dorsal aspect at five assessments across the goats′ first two lactations. Hoof conformation was assessed using five subjective scores (toe length, heel shape, fetlock shape, claw splay, and claw shape) and two objective measures (toe length ratio and claw splay distance). Following the training of two observers, high levels of inter and intra-reliability were achieved for both the subjective scores (>0.8 weighted kappa) and objective measures (>0.8 Lin′s concordance correlation coefficient). Two aspects of the subjectively assessed ordinal scores were compared with the objective measures with high levels of accuracy (>0.8). This suggests that the subjective scores may be a suitable alternative to more time-consuming objective measures when assessment is completed using photographs.

## 1. Introduction

Assessment of hoof conformation is important due to its recognized relationship with the biomechanical functionality of the hoof [1]. Hoof conformation refers to the physical dimensions and shape of the hoof. In dairy cows, desirable hoof conformational traits include a short toe and steeply angled hoof, a straight fetlock [2], an upright heel [3], and even claws [4], thus enabling even weight distribution between the medial and lateral claws of the hoof [5]. Poor hoof conformation is associated with an animal′s susceptibility to hoof lesions and lameness [4,6,7], decreased reproductive performance [8], reduced milk production [9], and a greater risk of being culled [10,11]. Therefore, accurate assessment of hoof conformation is imperative for the identification of at-risk animals.

Hoof conformation can be assessed using either subjective scores or objective measures. Aspects of the objective hoof conformation assessment described by Vermunt and Greenough [12] are often used in dairy cows [12,13,14]. Features assessed commonly include measurements of claw/sole length, heel height and dorsal wall length using calipers, and claw angle using an angle gauge or protractor. Claw/sole length is determined based on the length of the abaxial wall and bulb that are in contact with the floor [12,13]. Heel height is defined as the distance from the floor to the skin–horn junction [14,15], and dorsal wall length is measured as the distance from the tip of the toe to the dorsal skin–horn junction [12,14,16,17]. Claw angle is measured as the slope of the dorsal border of the claw with respect to the floor surface [14,15]. An animal with good conformation, will have even claw length, greater heel height, shorter dorsal wall length and greater claw angle [2,3,4]. However, it should be noted that the naming of the different objective measures can vary between authors, for instance, the dorsal wall length has previously been referred to as the toe length [15,18] or claw length [19].

Objective measures are suggested to provide superior assessments compared to subjective scores as they are accurate and repeatable [12], allowing for thorough assessment of hoof conformation traits. However, objective measures can involve some subjective judgement by the observer. For instance, concave dorsal hoof walls are reported in dairy heifers [20]; therefore, when measuring the angle of the claw, it results in the observer having to decide on the placement of the protractor. Bhardwaj et al. [13] reported intra-observer (repeatability) and inter-observer (reproducibility) reliability when assessing hoof conformation in sheep using the Vermunt and Greenough method. Bhardwaj et al. [13] concluded that due to difficulties in defining measurement points, claw angle and heel height were aspects of hoof conformation that were unreliable for measurement in sheep. Despite the possible difficulties in defining measurement points, inter- and intra-observer reliability are rarely reported in studies using objective measures of hoof conformation in dairy cows.

To our knowledge, there is only one previous study that has objectively measured hoof conformation in dairy goats [21], which also used the methodology described by Vermunt and Greenough [12]. Koluman and Göncü [21] did not report any validation to support the use of cow measurements in goats. Additionally, although the authors state that hooves were rescored to assess variance amongst observers, inter-observer reliability was not reported.

Subjective assessments of hoof conformation involve visual assessments to allocate a categorical score for particular aspects of conformation [2,6,11]. They are quick and easy to use, require no technical equipment, can allow assessment of a large number of animals, and are therefore commonly used for live animal scoring on the farm [22]. Subjective scoring systems have been used to assess a number of aspects of hoof conformation, such as abnormal overgrowth and splayed feet in sows [11]; misshaped hooves in sheep [6]; and heel height, toe overgrowth, and fetlock shape in cows [2,23]. In dairy goats, subjective scores of hoof overgrowth [24,25] and claw deformation have been reported [26]; however, no other aspects of hoof conformation have been subjectively assessed. Potential limitations of subjective scores are poor inter- and intra-observer reliability as they are affected by both the scoring system used and previous experience [22]. Therefore, intensive training is often required for high levels of reliability to be achieved using subjective methods of assessment [27].

It is important that reliability testing is conducted for conformation scoring systems to ensure that accurate and reliable results are obtained. Without evaluating repeatability and reproducibility, any conclusions made from the results may be misleading [23]. Additionally, assessments of hoof conformation need to be validated to ensure results are accurately indicating how the allocated scores relate to poor conformation. A way to validate a subjective assessment is to compare allocated subjective scores against objective measures. This has been carried out in pain and lameness assessments [28] and body condition scores in dairy cows [29]. However, validation of many hoof conformation assessment methods has not been reported. Therefore, the aims were to (1) develop a reliable method of assessing hoof conformation in dairy goats, and (2) compare two aspects of the subjective scoring assessment against corresponding objective measures as a means of validation.

## 2. Materials and Methods

This study was approved by AgResearch Ltd., Ruakura Animal Ethics Committee (#13478, approved 7 May 2015) as part of a large longitudinal study of dairy goat longevity. Sixteen commercial dairy goat farms in the Waikato region of New Zealand volunteered to participate (see Todd et al. 2019 for farm information; [30]). On 12 of the farms, the goats were permanently housed in barns and bedded on wood shavings. One farm provided the goats with access to outdoor pasture up to first kidding (assessment 2), but goats were permanently housed and bedded on wood shavings thereafter. On two farms, the goats were housed in barns and bedded on shavings, however, an outdoor area was provided for their adult goats once they were part of the milking herd. One farm housed the goats up to weaning and they were outdoors on pasture thereafter. All farms milked twice daily.

Farms were visited at five assessments throughout the goats′ first two lactations (2016–2017) (Table 1). As part of these visits, photographs of hooves were taken. The goats were all born in the previous season (May–August 2015) and were therefore of a similar age at the first assessment (mean ± SD: 8.0 ± 0.7 months of age). The first assessment was made near the time of first mating, at which point 1099 goats were still present in the longitudinal study; however, due to issues with hooves being dirty, poor photo quality, and missing goat identification, 1035 goats were included in the first assessment of the present study. By assessment 2, the goats had kidded and entered the milking herd; the number of goats contributing photographs decreased throughout the study due to culling and identification issues. Each farm′s housing and husbandry management protocol was maintained throughout the study, including their specific hoof management and trimming regimes.

### 2.1. Hoof Conformation Assessment

The hoof conformation assessment was adapted from subjective scores and objective measures previously reported for several species (Table 2). A digital camera (Canon Powershot, SX530) was used to take photographs of the left front and left hind hoof. For practicality and to reduce handling of the goats, only the left hooves were assessed. Photographs were taken in the yards outside of the milking parlor where goats were standing on a horizontal level concrete surface, which ensured they were bearing weight evenly on all four limbs. Two photographs per hoof were taken: (1) Lateral aspect and (2) dorsal aspect. Photographs were taken at approximately 50 cm from the goat, ensuring the hoof up to the knee/hock was in view. The hooves were photographed against a whiteboard, which had 2-cm scale markers along the vertical and horizontal edges to allow the objective measures to be calculated. 

**Figure 1:**
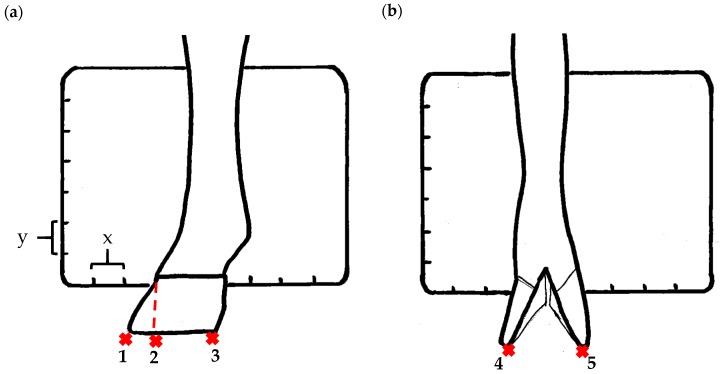
Methods to calculate objective measures of toe length ratio (**a**) and claw splay distance (**b**) using a developed R code and the 2-cm horizontal and vertical scale markers as reference points (x-distance and y-distance) for distance calibration. (**a**) A mark was placed on the photograph at the end of the toe (point 1), in line with the front edge of the coronet band (skin–horn junction of the hoof) (point 2), and at the back edge of the heel (point 3). The distance between point 1 and point 2 was divided by the distance between point 2 and point 3 to calculate the ratio. (**b**) A mark was placed on the photograph at the axial edge of the distal tip of both claws (points 4 and 5) to give the claw splay distance.

**Figure 2:**
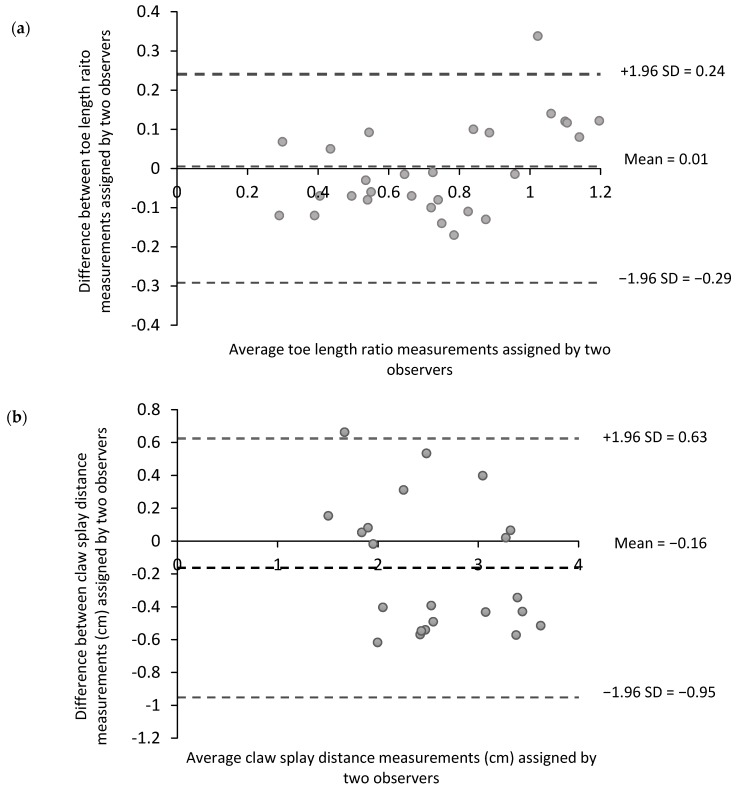
Bland–Altman plots showing the average of the two observers′ objective measurements against their difference. (**a**) Toe length ratio (*n* = 30 photographs) and (**b**) claw splay distance (*n* = 22 photographs) at training session 10. The middle line represents the estimated bias between the two observers, measured as the mean of the differences. The upper and lower dashed lines show limits of agreement (±1.96 SD of the observed differences).

The assessment included five subjective scores: (1) toe length, (2) heel shape, (3) fetlock shape, (4) claw splay, and (5) claw shape (Table 3 and Table 4). Each aspect was scored on a 3-point ordinal scale (0, 1, and 2), except for fetlock shape, which was scored on a binary scale (0 or 1); a 0 was ‘normal’ in all cases. Two objective measures were also made: (1) Toe length ratio (i.e., the toe length compared with the length of the rest of the hoof (Figure 1a) and (2) claw splay distance (i.e., distance between the axial edge of the distal tip of both claws (Figure 1b). Claw splay was scored, and claw splay distance measured, only when the claw shape was scored as a 0 (i.e., both claws were straight).

Two observers scored the photographs. Individual photographs were randomly allocated to each observer, ensuring that both observers scored photographs from each farm at each assessment. Observers completed scoring in a cyclical manner: A set of 20 photographs from one farm were completed and then the observer moved on to the next set to ensure photographs from several farms were scored on any given day. The subjective scoring and objective measures were performed in R 3.5.0 statistical software (R Core Team 2018, R Foundation for Statistical Computing, Vienna, Austria) [32]. An R code was developed using packages jpeg and tcltk2 to load and read the photographs, and packages zoo and latticeExtra for distance calibrations (see Supplementary File for a copy of the full R code used). The developed code streamlined the assignment of each subjective score at the same time as the objective measures were completed.

Using the developed code, a set of 20 photos were uploaded into R; the user firstly entered whether it was a lateral or dorsal aspect photograph they were viewing. A distance calibration was then completed using the scale bar marker on the whiteboard in the photographs. Four calibration points were selected on the scale bar. Two consecutive horizontal markers (x-distance) were firstly selected (cal1, cal2) and then two consecutive vertical markers (y-distance) were selected (cal3, cal4) (Figure 1a). The user input the width and height of the selected points as 2 cm, allowing the distance in pixels to be converted to a distance in centimeters. A linear regression was then fit for both the x-distance ((0, width) ~ intercept + slope *(cal1, cal2)) and the y-distance ((0, width) ~ intercept + slope *(cal3, cal4)). The estimated slopes and intercepts from the linear regressions for the x-distance and y-distance were then used to calibrate selected points on the hooves.

For the objectively measured toe length ratio, three points were selected on a lateral aspect hoof photograph; one point on the end of the toe (point 1), one point in line with the front edge of the coronet band (point 2), and one point at the back edge of the heel where the heel meets the ground (point 3) (Figure 1a). The distance between point 1 and point 2 was divided by the distance between point 2 and point 3 as follows:

(1) Toe length ratio=sqrt((x[2]− x[1])^2 + (y[2]− y[1])^2)sqrt((x[2]− x[3])^2 + (y[2]− y[3])^2),

where (x[2]–x[1]) is the calibrated difference of the x-position of point 2 on the hoof minus the x-position of point 1, and (y[2]–y[1]) is the calibrated difference of the y-position of point 2 on the hoof minus the y-position of point 1. Likewise, (x[2]–x[3]) is the calibrated difference of the x-position of point 2 on the hoof minus the x-position of point 3, and (y[2]–y[3]) is the calibrated difference of the y-position of point 2 on the hoof minus the y-position of point 3.

For the claw splay distance, two points were selected on a dorsal aspect hoof photograph; one on the axial side of the distal tip of both claws, with the medial claw (inside claw) selected first (point 1) (Figure 1b). These two points were calibrated as described above and then the distance between the two points was calculated as follows:

(2) Claw splay distance= sqrt((x[2] − x[1])^2 + (y[2] − y[1])^2),

where (x[2]–x[1]) is the calibrated difference of the x-position of point 2 on the hoof minus the x-position of point 1, and (y[2]–y[1]) is the calibrated difference of the y-position of point 2 on the hoof minus the y-position of point 1.

### 2.2. Inter and Intra-Observer Reliability 

Training involved scoring 400 photographs over 10 training sessions undertaken over a one-month period until an acceptable level of inter- and intra-observer reliability was achieved. A training session involved both observers independently scoring several photographs, and results were then compared and discussed before the next training session was conducted.

Of the 13,921 hoof photographs scored in total, observer 1 scored 7901 and observer 2 scored 6020. The number of photographs scored by each observer contained an equal balance of both lateral and dorsal aspect photographs. Throughout the photograph scoring, on-going inter-observer reliability tests were completed after both observers had scored approximately 400 photographs. This resulted in 15 inter-observer reliability tests being completed. Intra-observer reliability was tested by observers re-scoring 10% of photographs from each farm at each assessment.

For the subjectively scored aspects of hoof conformation (toe length, heel shape, fetlock shape, claw shape, claw splay), weighted kappa (*k_w_*) statistics were used to measure agreement. Acceptability was deemed as being above 0.8 (almost perfect agreement; [33]).

For the objectively measured aspects of hoof conformation (toe length ratio and claw splay distance), the Bland–Altman method was used to graphically assess agreement [34]. This involved plotting the average of the two observers′ measurements (x-axis) against their difference (y-axis), as well as the 95% confidence interval around the mean differences (±1.96 SD (standard deviation)). It is recommended that 95% of the data points on the Bland–Altman plot fall within ±1.96 SD of the mean difference [35]. Additionally, a Lin′s concordance correlation coefficient (CCC) [36] was calculated for the objective measures as this method contains measures of both accuracy and precision to determine how far the observed data deviate from the line of perfect concordance [36]. Acceptability of the CCC was deemed as being above 0.8 (high level of agreement; [37]).

For each inter-observer reliability test, if reliability went below a threshold of 0.8 for either *k_w_* or CCC, further training was completed to ensure reliability was 0.8 or above before scoring of the photographs could continue.

### 2.3. Comparison of Objective Measures and Subjective Ordinal Scores

Data processing and descriptive statistical analysis was performed using R 3.5.0 statistical package (R Core Team, 2018). The objective measures of the toe length ratio and claw splay distance were checked for outliers. If data points were 3 or more times the interquartile range away from the first and third quartile, they were considered outliers. There were 40 photographs identified as outliers for the toe length ratio and 5 photographs identified for the claw splay distance. One observer rescored these photographs, and if the original measurement was accurate, the data point remained in the data set. After rescoring, 34 outliers were deemed as accurate for the toe length ratio and 4 for the claw splay distance and thus remained in the data set.

To evaluate whether subjective scores were correctly assigned, thresholds were set for the toe length ratio as follows: If the ratio was <0.5 (length of toe was less than half of the length of the rest of the hoof), the score = 0; if the ratio was >0.5 and <1 (length of the toe was greater than half, but less than the full length of the rest of the hoof), the score = 1; and if the ratio >1 (length of the toe was greater than the full length of the rest of the hoof), the score = 2) (Table 3). Thresholds were set for the claw splay distance as follows: If the distance between claws was <4 cm, the score = 0; if the distance was >4 cm and <6 cm, the score was = 1; and if the distance was >6 cm, the score was = 2 (Table 4).

Contingency tables were produced to examine the assigned subjective scores for the toe length and claw splay to the actual scores (calculated using the above thresholds) for the front and hind hooves across all assessments and farms. An overall accuracy was calculated for each of the ordinal categories (0, 1, and 2) for the front and hind hooves. Accuracy was calculated at the level of each farm across the 5 assessments. Box plots were used to visually assess the consistency of scoring across the five assessments for the front and hind hooves.

Accuracy was calculated as follows using the number of true positive (TP), true negative (TN), false negative (FN), and false positive (FP) [38]:

(3) $$Accuracy\;=\;\frac{\left(TN+TP\right)}{\left(TN+TP+FN+FP\right)}\;=\;\frac{Number\;of\;correct\;assessments}{Number\;of\;all\;assessments}.$$

## 3. Results

### 3.1. Training

Over the 10 training sessions, inter-observer reliability increased. For the subjective scores over training sessions 1 to 4, *k_w_* ranged from 0.32 to 0.86 (median = 0.59, Q1: 0.46, Q3 = 0.73). Over training sessions 5 to 7, *k_w_* ranged from 0.53 to 0.88 (median = 0.71, Q1= 0.62, Q3 = 0.79). From sessions 8 to 10, *k_w_* was consistently over 0.8. For the objective measures, over training sessions 1 to 4, CCC ranged from 0.52 to 0.79 (median = 0.79, Q1 = 0.66, Q3 = 0.84) for the toe length ratio and 0.24 to 0.95 (median = 0.81, Q1 = 0.53, Q3 = 0.88) for the claw splay distance. Over training sessions 5 to 7, the CCC ranged from 0.79 to 0.91 (median = 0.79, Q1 = 0.73, Q3 = 0.85) for the toe length ratio and 0.82 to 0.89 (median = 0.85, Q1 = 0.84, Q3 = 0.87) for the claw splay distance. Over training sessions 8 to 10, the CCC ranged from 0.79 to 0.92 (median = 0.86, Q1 = 0.83, Q3 = 0.89) for the toe length ratio and 0.93 to 0.95 (median = 0.93, Q1 = 0.93, Q3 = 0.94) for the claw splay distance.

The Bland–Altman plots for the measures of the toe length ratio and claw splay distance showed a random scatter of points, with the majority of points falling within the limits of agreement (Figure 2).

### 3.2. Ongoing Inter-Observer Reliability 

Inter-observer reliability across the 15 reliability tests ranged from 0.63 to 1.00 (median: 0.81; Q1: 72; Q3: 91) (*k_w_*) for the subjective scores and 0.76 to 0.99 (median: 0.88; Q1: 82, Q3: 0.93) for the objective measures throughout the study (Table 5). At test 2 and 10, the CCC for the toe length ratio went below the 0.8 CCC threshold (0.79 and 0.76, respectively). At test 5, the claw splay score went below the 0.8 *k_w_* threshold (0.63), and at test 8, the claw shape went below the 0.8 *k_w_* threshold (0.71) (Table 5).

High levels of reliability were achieved for the fetlock shape subjective score; however, it should be noted that very few dipped fetlocks were recorded during the scoring of the lateral hoof photographs. A total of 186 were recorded out of 7058 lateral photographs (median: 33; Q1: 29, Q3: 37 dipped fetlocks per assessment).

### 3.3. Ongoing Intra-Observer Reliability 

Intra-observer reliability was consistently over 0.8 for the subjectively scored aspects (ranged from 0.82 to 1.00 (median: 0.91; Q1: 0.87; Q3: 0.96) (*k_w_*)) and the objectively measured aspects (ranged from 0.85 to 0.99 (median: 0.92; Q1: 0.89; Q3: 0.96) (CCC)) of hoof conformation.

### 3.4. Comparison of the Objective Measures and Subjective Scores

**Figure 3:**
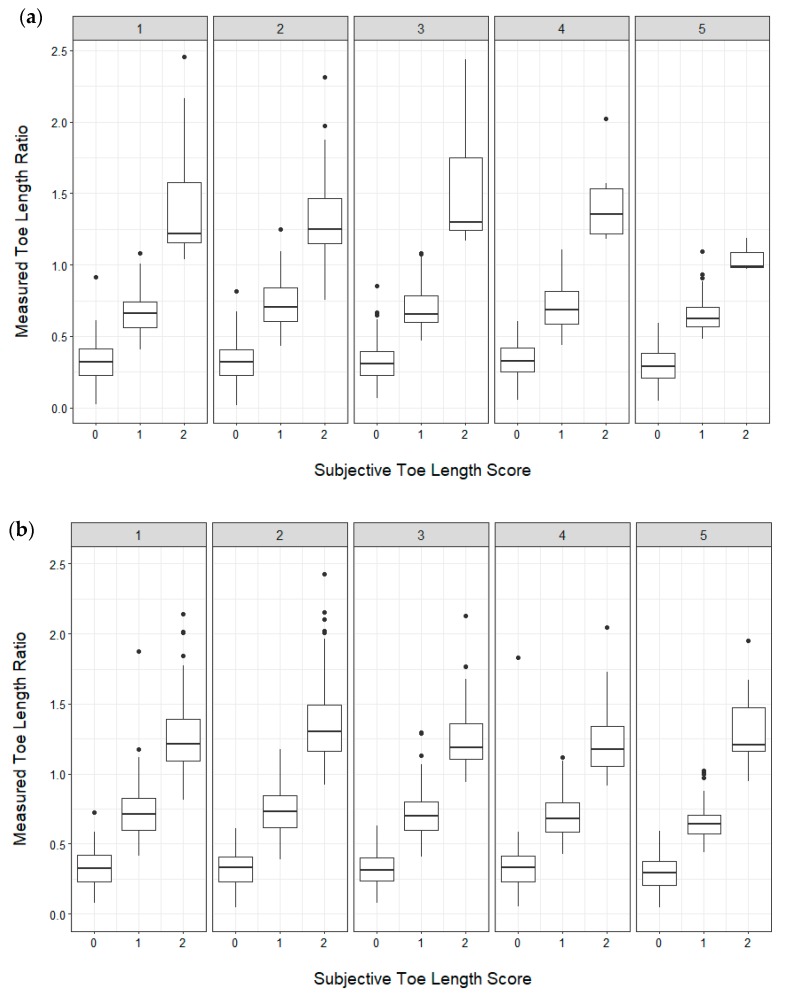
Box plots showing the distribution of the assigned toe length scores (0,1,2) and the measured toe length ratio (toe length measurement relative to the length of the rest of the hoof) across five assessments for the left front (**a**) and hind (**b**) hooves. Box plots show the 25th and 75th percentile (box), median (center line), and extreme values (whiskers). Possible outliers (dots) were checked to ensure they fell within three interquartile ranges away from the first and third quartile (*n* = 1035 contributing goats (median = 629, min = 573, Q1 = 576, Q3 = 791, max = 1035 contributing goats per assessment); *n* = 7058 total lateral hoof photographs (median = 1240, min = 1108, Q1 = 1130, Q3 = 1551, max = 2029 total front and hind photographs per assessment)).

**Figure 4:**
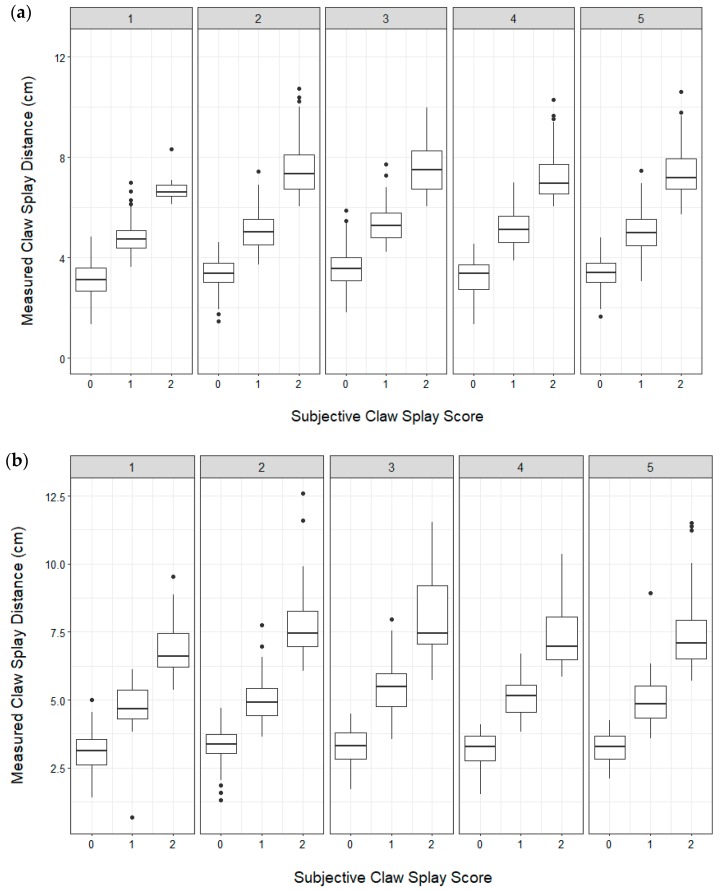
Box plots showing the distribution of assigned claw splay scores (0, 1, 2) and the measured claw splay distance (distance between the axial edge of the distal tip of both claws) across five assessments for the left front (**a**) and hind (**b**) hooves. Box plots show the 25th and 75th percentile (box), median (center line), and extreme values (whiskers). Claw splay was only scored if claws were not misshaped, therefore not all photographs/goats are included. Possible outliers (dots) were checked to ensure they fell within three interquartile ranges away from the first and third quartile (n = 1025 total number of goats that had at least 1 splay claw at any assessment (median = 511, min = 380, Q1 = 440, Q3 = 556, max = 758 contributing goats per assessment); *n* = 3579 total dorsal hoof photographs (median = 714, min = 486, Q1 = 600, Q3 = 738, max = 1041 total front and hind photographs per assessment)).

High levels of accuracy were achieved for the subjective assessments of the toe length and claw splay (>0.8) for each of the ordinal score categories when compared with the objective measures. The accuracy was the highest when assigning a score of 0 and was lower for scores 1 and 2 for both toe length (Table 6) and claw splay (Table 7). Scoring was relatively consistent across assessments (Figure 3 and Figure 4) and over farms. Over the farms accuracy for toe length score ranged from 0.90–0.96 for score 0 (median = 0.95, Q1 = 0.95, Q3 = 0.96), 0.89–0.95 for score 1 (median = 0.93, Q1 = 0.92, Q3 = 0.93), and 0.88–0.98 score 2 (median = 0.93, Q1 = 0.90, Q3 = 0.94). Over the farms accuracy for claw splay score ranged from 0.90–0.97 for score 0 (median = 0.94, Q1 = 0.94, Q3 = 0.95), 0.78–0.95 for score 1 (median: 0.90, Q1: 0.89, Q3: 0.93), and 0.86–0.98 score 2 (median = 0.92, Q1 = 0.89, Q3 = 0.98).

## 4. Discussion

The aim of this study was to develop a reliable method to assess hoof conformation in dairy goats. The results suggest that the assessment method developed is a suitable and reliable way to assess hoof conformation in dairy goats using photographs. After extensive training, both the subjective scores and objective measures were assessed reliably by the two observers. Two aspects of the subjective scores were compared with the corresponding objective measures and were found to be accurate. This suggests that the subjective scores, particularly the 0 and 2 scores, alone may be adequate to assess hoof conformation in dairy goats.

Toe length, as a proxy for hoof overgrowth, is the aspect of hoof conformation that has previously been focused on in dairy goats [24,25]. This is likely because hoof overgrowth is suggested to the be the most common cause of hoof deformation in goats [26,39]. However, other aspects of hoof conformation are also important due to the potential implications to the goat. For example, lower heel angles may significantly increase stress and deformation of the hoof capsule (horses; [40]), and misshaped claws can result in local pressure concentrations, resulting in tissue overloading and an increased risk of claw horn lesions (cows; [41]). Therefore, other aspects of conformation that were deemed as potentially impacting the welfare of the goat were also included in the current assessment, such as heel shape, fetlock shape, claw splay, and claw shape.

A potential limitation of subjective methods of hoof conformation assessment is the poor reliability between observers [18]. Previous subjective approaches to assess hoof conformation are commonly dichotomous (i.e., normal or abnormal; good or bad) [6,11]. This is likely because fewer scoring categories result in higher levels of agreement [42], due to less ambiguity. In the present study, high and consistent levels of reliability were achieved for the three-point ordinal subjective scores of toe length, heel shape, and fetlock shape; however, consistent with previous research, the middle score (1) had overlap with the others (0 or 2). It should be noted that very few instances of dipped fetlock were reported in the present study; nevertheless, it is important to include fetlock shape in hoof conformation assessments, as dipped fetlocks have the potential to increase tension of the suspensory apparatus in the lower leg and hoof (horses; [43]). However, work demonstrating this association in ruminants is lacking. The claw shape and claw splay subjective scores in the present study were less reliable and intermittently required further training. This training involved observers discussing the disagreements and completing further reliability tests. Assessments of the photographs did not continue until an agreement of over 0.8 between the observers was achieved. This ensured ongoing reliability in the following tests. When photographs were being taken, efforts were made to ensure that the goat was standing squarely and bearing weight on all four legs. However, care was also needed with the placement of the camera, particularly with the dorsal aspect view photographs. If the camera was not placed squarely in front of the hoof, the angle of the photograph may make it more difficult to accurately score. Therefore, this may explain why lower reliability was achieved for the claw shape and claw splay subjective scores.

Two aspects of hoof conformation, toe length and claw splay, could be both subjectively scored and objectively measured, allowing comparisons between the two methodologies. When comparing the subjective scores and objective measures of toe length and claw splay, the observers in the present study were more accurate at assigning a score of 0 compared to 1 or 2, resulting in some overlap when looking at hooves with borderline scores. This highlights why a dichotomous score of “good” vs “bad” is commonly used in hoof conformation assessments. However, acceptable levels of accuracy (>0.8) were still obtained for scores 1 and 2 and this may be due to the intensive training that was completed prior to assessment of the photographs. We caution other authors that if an accuracy level of over 0.8 is required, collapsing scores to a binary assessment may be required. It should be noted that heel height has also been previously objectively measured in hoof conformation assessments in sheep and dairy cows, however, a lower observer reliability than other measurements of hoof conformation have been reported [13,44]; therefore, in the present study, heel shape was assessed as a subjective score only.

The present study highlights the need for considerable training to ensure inter- and intra-observer reliability when scoring hoof conformation from photographs. Intensive training was required to attain initial reliability levels and then ongoing reliability checks were conducted to ensure any deviation between the observers scoring was quickly detected. In contrast, Murray et al. (1994) [23] used three or four categories to subjectively assess three aspects of hoof conformation in cattle and reported that the highest percentage agreement achieved between two trained observers was 66% [22]. In that study, training was undertaken by assessing 50 postmortem hooves collected from the abattoir while actual assessment was conducted on live animals in the milking parlor.

Repeatability (intra-observer variation) and reproducibility (inter-observer variation) are important when trying to validate a method for assessing hoof conformation. However, for many hoof conformation assessments, repeatability and reproducibility have not been established. For example, Gomez et al. (2015) [14] evaluated the hoof conformation of 644 dairy cow heifers. However, all measurements were completed by one observer and no intra-observer reliability testing was reported. Intra-observer reliability is commonly more consistent than inter-observer reliability [23,45]. This is supported by the findings from the present study where intra-observer reliability was consistently above the 0.8 threshold for both *k_w_* and CCC. However, variance within an observer still needs to be reported. It is difficult to make definitive conclusions from studies where no evidence is provided to determine whether the method is repeatable or reproducible.

Hoof conformation has previously been objectively assessed using photographs with scale markers included for other species [31,46], and with similar methods used in the present study. With the methodology used, the objective measures used in the present study would not be possible to apply on live animals; thus, their use is restricted on farm. Additionally, for objective measures to be completed on farm, animals are often restrained (goats; [21]) using a crush and their hooves are tied (cows; [15]) or on a tilt table (cows; [47]). Furthermore, lifting and tying hooves for objective measures to be completed may not give a true assessment of hoof conformation. The shape of the hoof is influenced by weight bearing and load [48]; therefore, if the animal is not weight bearing on a limb, it may not accurately reflect the animal′s true conformation. In the present study, the use of photographs to obtain objective measures reduced the need for such restraint, and ensured the goats were weight bearing to give a true reflection of conformation.

The objective measure for the claw splay distance was consistently reliable throughout the scoring of the hoof photographs. There were two occasions when the reliability for the objective measure of the toe length ratio went below 0.8. This may have been due to difficulties in placing a point on the hoof in line with the front edge of the coronet band, especially if the hooves were particularly hairy or dirty. Due to time restrictions around milking and attempting to minimize the amount of time the goats were out of their pens, it was not feasible for the hooves to be washed. However, if possible, we recommend cleaning of the hooves prior to photographs being taken to improve reliability. As the reliability for the subjective score for toe length was consistently high throughout the assessments, it suggests that the subjective score is more appropriate to use rather than the time-consuming objective measure; however, this needs to be validated on farm.

## 5. Conclusions

We successfully developed a reliable method for assessing hoof conformation in dairy goats using photographs. Two aspects of hoof conformation that were subjectively assessed were validated by the comparison of the subjective scores with objective measures. The use of photographs with scale markers allowed for objective measures to be completed; however, this was time consuming and required technical equipment. As two of the subjective scores were shown to correspond to objective measures, they are suitable methods for conformation assessment. High levels of accuracy and reliability (>0.8) were achieved on the photographs in this study; if higher levels were required, than collapsing the scores into a binary method should be considered. Nevertheless, further work is required to test the reliability and practicality of subjective hoof conformation assessment on live animals and to determine if it is applicable in an on-farm setting.

## Figures and Tables

**Table 1:** Stage of production, age (mean ± SD (months)) of the goats, the number of farms visited, the number of goats, and the number of hoof photographs scored at each of the five assessments across the first two lactations.

Assessment	Stage of Production	Age	Number of Farms *	Number of Goats Contributing Photos ^†^	Number of Lateral Aspect Photographs **		Number of Dorsal Aspect Photographs **	
					Front	Hind	Front	Hind
1	First mating	8.0 ± 0.70	16	1035	1018	1011	998	990
2	Start of first lactation	14.8 ± 0.86	15	791	782	769	760	769
3	End of first lactation	21.9 ± 0.70	13	573	561	547	530	536
4	Start of second lactation	29.1 ± 1.00	13	576	566	564	540	547
5	End of second lactation	34.1 ± 0.90	13	629	624	616	594	599

* All 16 farms were included at assessment 1. Issues with photo quality and hoof cleanliness prevented scoring on one farm on assessment 2 and two farms on assessments 3 and 4. At assessment 5, farm visits could not take place on two of the farms and one farm was withdrawn from the trial (note: these are not the same farms missing at assessments 3 and 4, therefore goat numbers differ). ^†^ Goat numbers declined as the trial progressed due to culling and ID issues. ** Not all the goats′ photos were scored due to hooves being too dirty, or the photographs being of insufficient quality (e.g., blurry or too dark) for observers to accurately score.

**Table 2:** Aspects of hoof conformation adapted from previous subjective and objective assessments to create the current approach of assessment for dairy goats.

Species	Assessment Type	Aspects of Hoof Conformation	References
Cows	Objective	Toe length, heel height	[15,18,31]
Sheep	Subjective	Shape of hoof	[6]
Sows	Subjective	Abnormal hoof growth, splayed feet, dipped pastern/fetlock	[11]
Goats	Subjective	Hoof overgrowth	[24,25,26]

**Table 3:** Hoof conformation aspects subjectively assessed from photographs taken of the lateral aspect of the left front and left hind hooves of dairy goats across their first 2 lactations, at up to 16 farms and 5 assessments: (1) First mating, (2) start of first lactation, (3) end of first lactation, (4) start of second lactation, and (5) end of second lactation (*n* = 1035 contributing goats (median = 629, min = 573, Q1 = 576, Q3 = 791, max = 1035 contributing goats per assessment); *n* = 7058 total lateral hoof photographs (median = 1240, min = 1108, Q1 = 1130, Q3 = 1551, max = 2029 total of front and hind photographs per assessment); not all the goats′ photos were scored due to hooves being too dirty or the photographs being of insufficient quality (e.g., blurry or too dark) for observers to accurately score).

Hoof Aspect	Ordinal Score		
	0	1	2
Toe length	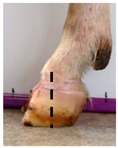	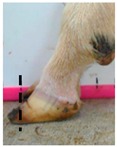	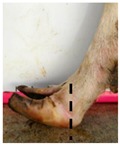
	**Toe is not overgrown** Length of the toe is less than half of the length of rest of the hoof	**Toe is moderately overgrown** Length of the toe is greater than half, but less than the full length of the rest of the hoof	**Toe is severely overgrown** Length of the toe is greater than the full length of the rest of the hoof
Heel shape	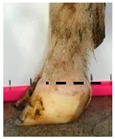	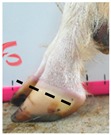	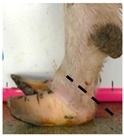
	**Heel is upright** Not walking on heel, coronet band parallel to ground	**Heel is moderately dipped** Not walking on heel, but coronet band is angled towards the ground	**Heel is severely dipped** Walking on heel, coronet band angled sharply towards the ground
Fetlock shape *	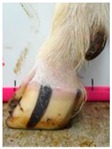	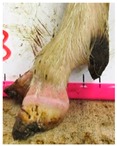	
	**Fetlock is upright and straight**	**Fetlock is dipped towards the ground** Bony lump on pastern may be apparent	

* Fetlock scored as binary 0 or 1.

**Table 4:** Hoof conformation aspects subjectively assessed from photographs taken of the dorsal aspect of the left front and left hind hooves of dairy goats across their first 2 lactations, at up to 16 farms and 5 assessments: (1) First mating, (2) start of first lactation, (3) end of first lactation, (4) start of second lactation, and (5) end of second lactation (*n* = 1035 contributing goats (median = 629, min = 573, Q1 = 576, Q3 = 791, max = 1035 contributing goats per assessment); (*n* = 6863 total dorsal photographs (median = 1193, min = 1066, Q1 = 1087, Q3 = 1529, max = 1988 total of front and hind photographs per assessment); not all the goats′ photos were scored due to hooves being too dirty, or the photographs being of insufficient quality (e.g., blurry or too dark) for observers to accurately score).

Hoof Aspect	Ordinal Score		
	0	1	2
Claw shape	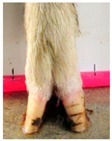	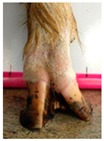	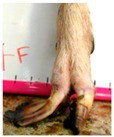
	**Both claws are straight**	**One claw is bent/twisted** either away or towards the midline of the hoof	**Both claws are bent/twisted** either away or towards the midline of the hoof
Claw splay *	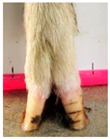	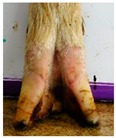	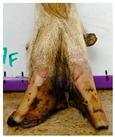
	**Claws are not splayed** the distance between the axial edge of the distal tip of both claws are approximately <2 horizontal marks on the whiteboard	**Claws are moderately splayed** the distance between the axial edge of the distal tip of both claws are approximately >2 and <3 marks on the whiteboard	**Claws are severely splayed** the distance between the axial edge of the distal tip of both claws are >3 marks on the whiteboard

* Claw splay only scored if claw shape scored as 0.

**Table 5:** Results of 15 inter-observer reliability tests completed by the two observers for the subjective scores and objective measures of the hoof conformation assessment.

Test	Subjective Scores (Weighted Kappa (95% CI))					Objective Measures (Lin′s Concordance Coefficient (95% CI))	
	Toe Length	Heel	Fetlock	Claw Shape	Claw Splay	Toe Length Ratio	Claw Splay Distance
1	0.84 (0.72–1.00)	1.00 (1.00–1.00)	0.83 (0.73–1.00)	1.00 (1.00–1.00)	1.00 (1.00–1.00)	0.86 (0.70–0.97)	0.97 (0.87–0.99)
2	0.91 (0.73–1.00)	0.92 (0.77–1.00)	0.83 (0.73–1.00)	1.00 (1.00–1.00)	0.90 (0.71–1.00)	0.79 (0.46–0.93) *	0.97 (0.81–1.00)
3	0.83 (0.70–1.00)	0.85 (0.78–1.00)	0.87 (0.61–1.00)	0.92 (0.76–1.00)	1.00 (1.00–1.00)	0.80 (0.62–0.93)	0.99 (0.94–1.00)
4	0.83 (0.69–1.00)	0.83 (0.71–1.00)	1.00 (1.00–1.00)	0.82 (0.72–1.00)	1.00 (1.00–1.00)	0.94 (0.82–0.98)	0.89 (0.79–0.99)
5	0.91 (0.75–1.00)	1.00 (1.00–1.00)	1.00 (1.00–1.00)	0.87 (0.71–1.00)	0.63 (0.39–1.00) *	0.83 (0.67–0.91)	0.88 (0.63–0.96)
6	1.00 (1.00–1.00)	0.89 (0.68–1.00)	1.00 (1.00–1.00)	0.88 (0.64–1.00)	0.82 (0.60–1.00)	0.84 (0.64–0.94)	0.99 (0.95–1.00)
7	1.00 (1.00–1.00)	1.00 (1.00–1.00)	1.00 (1.00–1.00)	0.85 (0.68–1.00)	0.84 (0.72–1.00)	0.95 (0.82–0.98)	0.99 (0.86–0.99)
8	1.00 (1.00–1.00)	0.88 (0.77–1.00)	1.00 (1.00–1.00)	0.71 (0.49–1.00) *	0.86 (0.73–1.00)	0.80 (0.53–0.92)	0.97 (0.90–0.98)
9	0.88 (0.65–1.00)	0.89 (0.74–1.00)	1.00 (1.00–1.00)	0.88 (0.65–1.00)	1.00 (1.00–1.00)	0.97 (0.92–0.99)	0.97 (0.81–0.99)
10	0.87 (0.72–1.00)	0.95 (0.88–1.00)	1.00 (1.00–1.00)	0.87 (0.71–1.00)	0.83 (0.59–1.00)	0.76 (0.64–0.84) *	0.93 (0.83–0.97)
11	0.88 (0.74–1.00)	1.00 (1.00–1.00)	1.00 (1.00–1.00)	0.84 (0.74–1.00)	1.00 (1.00–1.00)	0.81 (0.69–0.92)	0.91 (0.78–0.97)
12	0.89 (0.78–1.00)	1.00 (1.00–1.00)	1.00 (1.00–1.00)	0.86 (0.81–1.00)	1.00 (1.00–1.00)	0.84 (0.66–0.95)	0.95 (0.73–0.99)
13	0.89 (0.78–1.00)	0.88 (0.75–1.00)	1.00 (1.00–1.00)	1.00 (1.00–1.00)	1.00 (1.00–1.00)	0.89 (0.72–0.96)	0.96 (0.84–1.00)
14	0.87 (0.72–1.00)	0.96 (0.88–1.00)	1.00 (1.00–1.00)	0.86 (0.71–1.00)	0.83 (0.79–1.00)	0.86 (0.67–0.94)	0.93 (0.79–0.98)
15	0.92 (0.77–1.00)	0.93 (0.80–1.00)	1.00 (1.00–1.00)	0.92 (0.75–1.00)	0.81 (0.74–1.00)	0.94 (0.88–0.97)	0.97 (0.88–1.00)

* Occasions where reliability went below 0.8.

**Table 6:** The number of correctly assigned scores (in bold), the number of incorrectly assigned scores, and accuracy for toe length ordinal scores (0, 1, and 2) for the left front and hind hooves as compared with the measured toe length ratio (toe length (end of the toe to the abaxial edge of hoof in line with the front edge of the coronet band) compared with the length of the rest of the hoof (abaxial edge of hoof in line with the front edge of the coronet band to the back edge of the heel)). Scored from hoof photographs taken from a lateral aspect at up to 16 farms and 5 assessments (*n* = 1035 contributing goats (median = 629, min = 573, Q1 = 576, Q3 = 791, max = 1035 contributing goats per assessment); *n* = 7058 total lateral hoof photographs (median = 1240, min = 1108, Q1 = 1130, Q3 = 1551, max = 2029 total front and hind photographs per assessment)).

Assigned Scores	Front Hooves				Hind Hooves			
	Actual Toe Length Scores ^a^			Accuracy	Actual Toe Length Scores ^a^			Accuracy
	0	1	2		0	1	2	
0	**2359**	148	0	0.93	**1586**	80	1	0.96
	**(98.6%)**	(15.1%)	(0.0%)		**(96.0%)**	(5.9%)	(0.2%)	
1	34	**808**	33	0.91	63	**1247**	53	0.93
	(1.4%)	**(82.3%)**	(22.9%)		(4.0%)	**(92.2%)**	(11.8%)	
2	0	5	**111**	0.88	0	25	**395**	0.94
	(0.0%)	(0.6%)	**(77.1%)**		(0.0%)	(1.8%)	**(88.0%)**	
Total scores	2393	981	144		1649	1352	449	

^a^ Toe length scores: (0) Toe is not overgrown—The length of the toe is less than half of the rest of the hoof, (1) Toe is moderately overgrown—The length of the toe is greater than half, but less than the full length of the hoof, (2) Toe is severely overgrown—The length of the toe is greater than the full length of the rest of the hoof. Actual scores were calculated using the measured toe length ratios. If the ratio was <0.5, the score = 0; if the ratio was >0.5 and <1, the score = 1; and if the ratio was >1, the score = 2.

**Table 7:** The number of correctly assigned scores (in bold), the number of incorrectly assigned scores, and accuracy for claw splay ordinal scores (0, 1, and 2) for the left front and hind hooves as compared with the measured claw splay distance. Scored from hoof photographs taken from a dorsal aspect at up to 16 farms and 5 assessments. Claw splay was only scored if claws were not misshaped, therefore not all photographs/goats are included (*n* = 1025 total number of goats that had at least 1 splay claw at any assessment (median = 511, min = 380, Q1 = 440, Q3 = 556, max = 758 contributing goats per assessment); *n* = 3579 total dorsal hoof photographs (median = 714, min = 486, Q1 = 600, Q3 = 738, max = 1041 total front and hind photographs per assessment)).

Assigned Scores	Front Hooves				Hind Hooves			
	Actual Claw Splay Scores ^a^			Accuracy	Actual Claw Splay Scores ^a^			Accuracy
	0	1	2		0	1	2	
0	**809**	116	0	0.95	**548**	60	0	0.95
	**(97.8%)**	(12.7%)	(0.0%)		**(96.3%)**	(11.0%)	(0.0%)	
1	18	**795**	68	0.90	21	**481**	45	0.90
	(2.2%)	**(87.2%)**	(17.0%)		(3.7%)	**(87.9%)**	(15.8%)	
2	0	1	**332**	0.91	0	6	**239**	0.92
	(0.0%)	(0.1%)	**(83.0%)**		(0.0%)	(1.1%)	**(84.2%)**	
Total scores	827	912	400		569	547	284	

^a^ Actual scores were calculated using the measured claw splay distance. If the distance was <4 cm, the score = 0; if the distance was >4 cm and <6 cm, the score = 1; and if the distance was >6 cm, the score = 2.

## Supplementary Materials

Full copy of the R code used. The following are available online at https://www.mdpi.com/2076-2615/9/11/973/s1.
